# A New Validated Potentiometric Method for Sulfite Assay in Beverages Using Cobalt(II) Phthalocyanine as a Sensory Recognition Element

**DOI:** 10.3390/molecules25133076

**Published:** 2020-07-06

**Authors:** Saad S. M. Hassan, Ayman H. Kamel, Abd El-Galil E. Amr, Hisham S. M. Abd-Rabboh, Mohamed A. Al-Omar, Elsayed A. Elsayed

**Affiliations:** 1 Department of Chemistry, Faculty of Science, Ain Shams University, Cairo 11566, Egypt; 2 Pharmaceutical Chemistry Department, College of Pharmacy, King Saud University, Riyadh 11451, Saudi Arabia; malomar1@ksu.edu.sa; 3 Applied Organic Chemistry Department, National Research Center, Dokki 12622, Giza, Egypt; 4 Chemistry Department, Faculty of Science, King Khalid University, P.O. Box 9004, Abha 61413, Saudi Arabia; hasalah@hotmail.com; 5 Zoology Department, Faculty of Science, King Saud University, Riyadh 11451, Saudi Arabia; eaelsayed@ksu.edu.sa; 6 Chemistry of Natural and Microbial Products Department, National Research Centre, Dokki 12622, Cairo, Egypt

**Keywords:** sulfite measurements, potentiometry, sulfite sensor, beverages, flow injection analysis, method validation

## Abstract

A simple potentiometric sensor is described for accurate, precise, and rapid determination of sulfite additives in beverages. The sensor is based on the use of cobalt phthalocyanine as a recognition material, dispersed in a plasticized poly(vinyl chloride) matrix membrane. *o*-Nitrophenyl octyl ether (*o*-NPOE) as a membrane solvent and tri-dodecylmethyl- ammonium chloride (TDMAC) as ion discriminators are used as membrane additives. Under the optimized conditions, sulfite ion is accurately and precisely measured under batch and flow injection modes of analysis. The sensor exhibits fast and linear response for 1.0 × 10^−2^–1.0 × 10^−6^ M (800–0.08 µg/mL) and 1.0 × 10^−1^–5.0 × 10^−5^ M (8000–4 µg/mL) sulfite with Nernstian slopes of −27.4 ± 0.3 and −23.7 ± 0.6 mV/concentration decade under static and hydrodynamic modes of operation, respectively. Results in good agreement with the standard iodometric method are obtained.Validation of the assay method is examined in details including precision, accuracy, bias, trueness, repeatability, reproducibility, and uncertainty and good performance characteristics of the method are obtained. The sensor response is stable over the pH range of 5 to 7 without any significant interference from most common anions. The advantages offered by the proposed sensor (i.e., wide range of assay, high accuracy and precision, low detection limit, reasonable selectivity, long term response stability, fast response, and long life span and absence of any sample pretreatment steps) suggest its use in the quality control/quality assurance routine tests in beverages industries, toxicological laboratories and by inspection authorities.

## 1. Introduction

Sulfite ions have been widely used for centuries as preservative for food, beverages, and pharmaceutical products [1,2]. It is added to prevent oxidation, enzymatic and non-enzymatic browning, and bacterial growth [3]. As sulfite is considered as a hazard to human health, especially for persons with sulfite oxidase deficiency disease, the use of sulfite in foodstuff has to be controlled. The Food and Drug Administration, USFDA, has established 10 ppm as the threshold for declaration of sulfite in the labeling of food, non-alcoholic beverages, and wine products [4,5]. Therefore, the development of a sensitive, selective, fast, and cost effective method for the determination of sulfite is very important and highly demanded for food quality control and quality assurance.

Previously described analytical methods for the determination of sulfite include spectrophotometry based on reactions with formaldehyde-pararosaniline [6], methyl red [7], and diaquacobyrinic acid heptamethyl ester (diaquacobester, DACbs) [8]. Spectrofluorimetric methods for the determination of sulfite include the use of tetra-substituted amino aluminum phthalocyanine [9] rhodamine B hydrazide [10], and N-(9-acridinyl) maleimide (NAM) [11]. Indirect methods for spectrophotometric quantification of sulfite in beverageshave been also suggested [12,13]. Chemiluminescence (CL) measurements have been described using riboflavin phosphate or brilliant sulfaflavine [14], luminol [15], and riboflavin phosphate [16]. Phosphorimetric assay of sulfite has been described using Pt(II) coproporphyrin/bovine serum albumin [17].

Chromatographic methods for sulfite analysis have been widely used including ion chromatography [18], gas chromatography [19], liquid chromatography [20,21], zone electrophoresis [22] and ion exclusion chromatography [23]. Enzymatic assays using sulfite oxidase enzyme, supported on an oxygen sensor, and following the decrease in dissolved oxygen concentration [4,24,25] with an H_2_O_2_ sensor or Clark-type oxygen electrode. An amperometric sulfite oxidase/cytochrome c-based sensor has been also used [26]. However, most of the previously described methods and techniques are not selective, need tedious sample pretreatment steps, are time consuming, and are not sensitive enough to determine low concentrations of sulfite in real food samples.

Electrochemical techniques including amperometry and voltammetry have been described [27,28,29,30,31]. With the hallmarks of high sensitivity, selectivity, easy operation, and a variety of probes available, potentiometric sensors are a good alternative to sulfite detection. Ion-selective electrodes (ISEs) that contain selectivity carriers (i.e., ionophores) have been used widely to detect different types of ions in medical, environmental, and industrial analyzes [32,33,34,35,36,37]. On the other hand, little is known about the use of potentiometric sensors for sulfite quantitation. Potentiometric sulfite sensors with polymeric membranes incorporating mercury(II) diethydithiocarbamate [38], guanidinium ionophore [39], and solid-state membrane sensor based on titanium phosphate epoxy have been developed and used for manual and flow injection analysis of sulfite [40]. On the other hand, no commercial sensors are available in the market, so far, for sulfite quantification.

In this contribution, a simple, fast, and sensitive potentiometric sensor for sulfite determination under static and hydrodynamic modes of operation based on the use of cobalt phthalocyanine as an efficient sulfite recognition sensing reagent is described. This macrocyclic reagent is chemically stable, environmentally friendly, non-toxic, exhibits physically interesting electrochemical properties for selective sulfite sensing. A PVC membrane sensor based on cobalt phthalocyanine is developed, characterized and used under static and hydrodynamic modes of operation for sulfite measurements in beverages. The sensor is suitable for quality control/quality assurance in beverage industry as it offers low detection limit, a wide linear response range, good selectivity, high accuracy, long term stability, and fast response time with a low fabrication cost.

## 2. Results and Discussions

**Figure 1:**
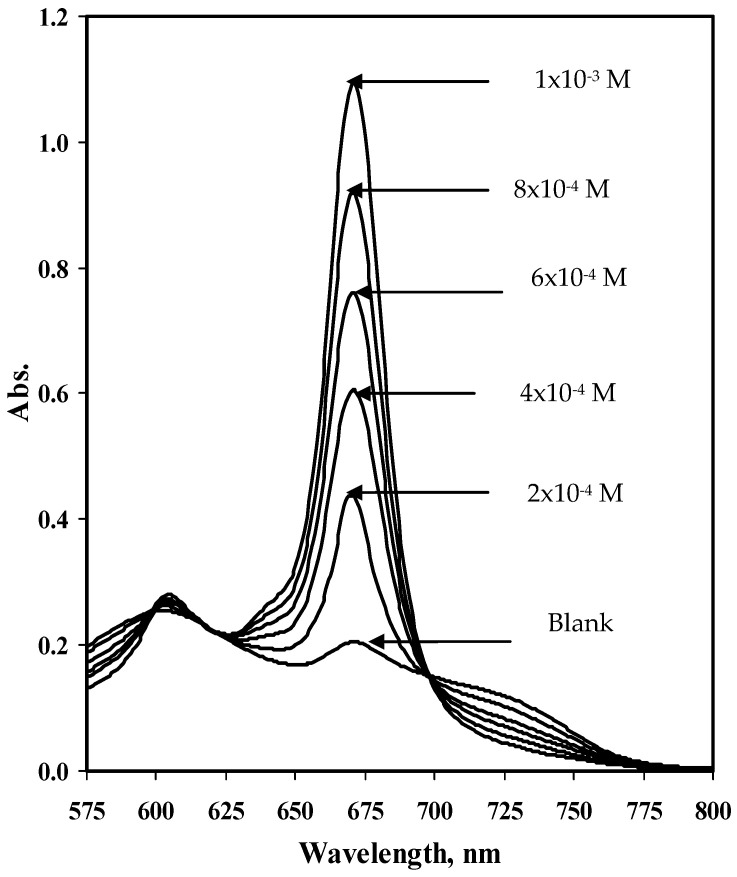
Absorption spectra of sulfite calibrants in the range of 2 × 10^−4^ to 1 × 10^−3^ M after the addition of 1 mL of 0.1 mg/mL CoPC in DMSO.

Cobalt phthalocyanine (CoPc), a *p*-type organic semiconductor with an 18 *π*-electron system, was examined as a potentiometric recognition ionophore for selective and sensitive potentiometric determination of sulfite ion. A preliminary investigation was conducted by testing the interaction of sulfite ion with a solution of Co(II)-phthalocyanine. Cobalt phthalocyanine was completely insoluble in aqueous solutions but readily dissolves in dimethyl sulfoxide (DMSO), tetrahydrofuran (THF), and dioxane solvents, displaying absorption spectrum in the visible region with two maxima. Solutions of cobalt phthalocyanine in tetrahydrofuran and dioxane were unstable because the absorbance at these two wavelengths rapidly decreased with time. Dimethyl sulfoxide (DMSO) showed good dissolution properties and long-term color stability of cobalt phthalocyanine. The absorption spectrum of Co (II)-phthalocyanine in DMSO solution displayed two absorption peaks at 620 and 666 nm (Figure 1).

**Figure 2:**
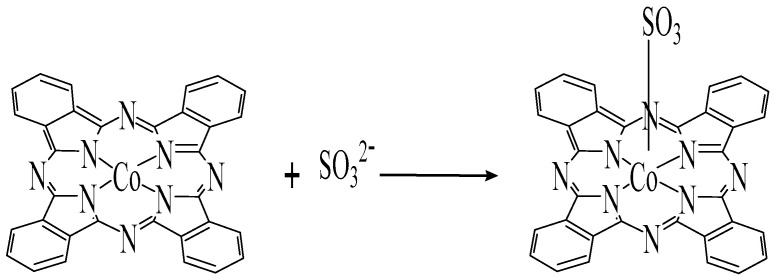
Ligation of sulfite ion to Co(II)-phthalocyanine complex.

Addition of sulfite ions to the Co (II)-complex solution caused a slight decrease in the peak intensity at 620 nm and a slight bathochromic shift of the 666 nm peak to 670 nm. The last peak was sensitive and linearly increased in absorbance with the increase of sulfite concentration over the range 16 to 80 µg/mL, probably due to ligation of the sulfite ion to one of the axial position on the Co(II)-complex with subsequent oxidation of Co(II) to Co(III), Figure 2. Although a Co(II)-phthalocyanine was used to prepare these ISE membranes, the response mechanism can be explained as a charged-carrier mechanism, which was speculated to be due to oxidation of the metal center to Co(III).

It has been reported that sulfite ion coordinates to iron and cobalt phthalocyanines during the electro catalytic oxidation of sulfur dioxide by these complexes [41]. Ligation of sulfite to Co(II)-tetrasulfophthalocyanine in organic solvents has been also demonstrated [42].

Common interfering anions (e.g., nitrite, nitrate, chloride, fluoride, azide, sulfate, phosphate, acetate, and thiocyanate ions) had no significant effect on the absorption spectrum compared to the blank. Addition of 50–100-fold excess of these ions did not contribute by more than 2% error. These data provide a clear evidence for the reasonable recognition characteristics and good sensitivity and selectivity of the proposed reagent towards sulfite ion over the most common interfering anions.

### 2.1. Potentiometric Recognition of Sulfite

**Figure 3:**
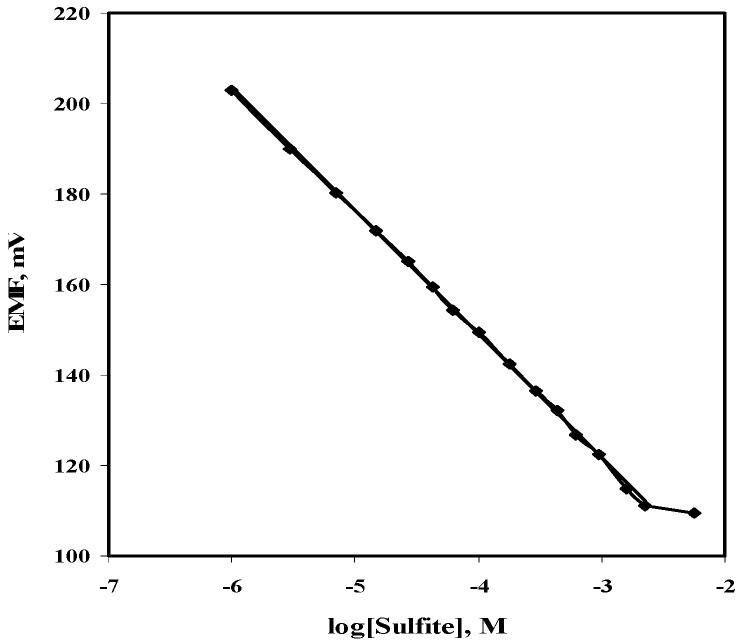
Calibration plot of sulfite ion using [CoPC/TDMA^+^/*o*-NPOE] PVC membrane-based sulfite sensor in a mixture of 10^−2^ M acetate buffer of pH 5 and 10^−3^ M NaCl as a background solution.

A polymeric membrane potentiometric sensor consisting of cobalt phthalocyanine as an ionophore, dioctylphthalate (DOP) or ortho-nitrophenyloctylether (*o*-NPOE) as a plasticizer, and PVC as polymeric matrix in the optimum ratio of (1.0:69:30), respectively, was prepared and tested as a sulfite ion sensor. Effects of the membrane plasticizer, background solution, and membrane additives were tested. A membrane incorporating DOP plasticizer (low dielectric constant, ε = 7) was first tested. A calibration graph made in 10^−3^ M NaCl background solution as an ionic strength adjustor (ISA) showed a linear potential response over the concentration range of 4.5 × 10^−5^ to 1.1 × 10^−3^ M sulfite (3.6–88 µg/mL) with a Nernstian slope of−25.5 ± 0.2 mV/decade and a detection limit of 2.0 × 10^−5^ M (1.6 µg/mL) (Figure 3). Calibration parameters in pure aqueous media were inferior to those obtained in the presence sodium chloride background.

The effect of incorporating aniondiscriminator as a membrane additive, such as tridodecylmethylammonium chloride (TDMAC) in the membrane phase of the sensor slightly improved the performance characteristics of the sensor (linear range, slope and detection limit of the calibration plot). The calibration slope became −26.8 mV/decade and the limit of detection reached 1.4 × 10^−5^. Membranes containing *o*-nitrophenyloctyl ether plasticizer, *o*-NPOE (high dielectric constant, ε = 23) were next examined. A typical calibration curve for the sulfite sensor incorporating CoPC/TDMA^+^/*o*-NPOE in a background of 10^−2^ M acetate buffer of pH 5, and 10^−3^ M NaCl is shown in Figure 3. The linear range increased to cover the range of 1.0 × 10^−6^ to 2.2 × 10^−3^ M (0.08–176 µg/mL) with a better detection limit of 1.0 × 10^−6^ M (0.08 µg/mL) and a calibration slope of –27.4 mV/decade of sulfite concentration. Table 1 summarizes the different performance characteristics of the sulfite sensor under the optimized conditions. All subsequent experiments were made with this sensor.

Table 1 Performance characteristics of the [CoPC/TDMA^+^/o-NPOE] sulfite PVC membrane sensor in 10^−2^ M acetate buffer and 10^−3^ M NaCl (pH 5) under batch and FIA modes of operation.

### 2.2. Effect of pH

**Figure 4:**
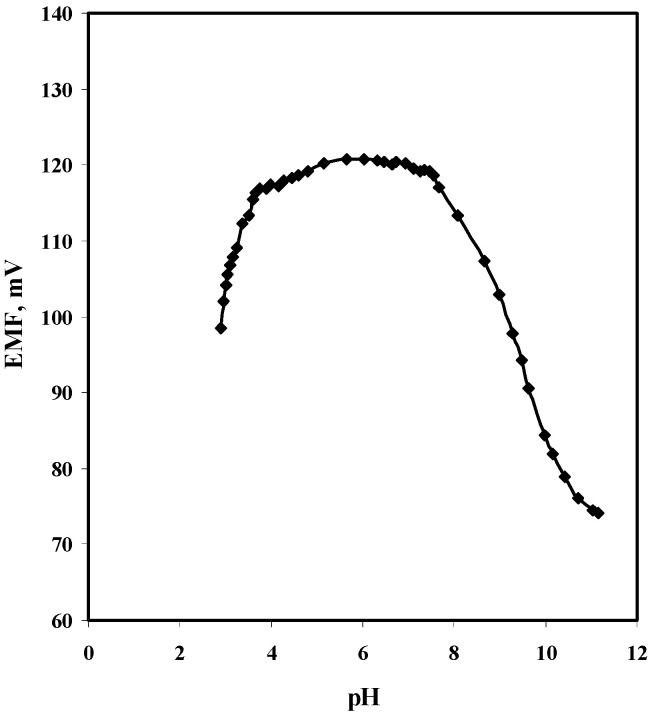
Effect of pH on the potentiometric response of [CoPC/TDMA^+^/*o*-NPOE] PVC membrane based sulfite sensor using 10^−3^ M sulfite solution.

The effect of pH on the potentiometric response of the sulfite sensor was evaluated by changing the pH of 1.0 × 10^−3^ M $SO_{3}^{2-}$ solution in a 1.0 × 10^−3^ M NaCl background from pH 3 to 11 by adding aliquots of concentrated sodium hydroxide and/or acetic acid solutions. The corresponding mV readings at each pH value were recorded after each addition. Figure 4 shows an independence of the sensor’s response for sulfite ions over the pH range 5 to 7. Above and below this range, the sensor potential was sharply decreased. At high pH values of >8, the sensor responded to OH^−^ ion, while at low pH values of < 3, the sensor responded to the monovalent HSO_3_^−^(*pKa_1_* = 1.89 and *pKa_2_* = 7.21 at 25 °C). A 10^−2^ M acetate buffer solution of pH 5, containing 10^−3^ M NaCl was used as a background solution and used for all subsequent measurements.

### 2.3. Potentiometric Selectivity of CoPC/TDMAC-based Sulfite Sensor

The potentiometric selectivity coefficients *(K^Pot^_Sulfite,J_)* of the sulfite sensor were measured according to the IUPAC guidelines using the fixed interfering method (FIM) [43]. In this method, 1 mL of 1.0 × 10^−2^ M of the interfering ion solution was added to 9.0 mL of a 1:1 mix of 1.0 × 10^−3^ M NaCl and 1.0 × 10^−3^ M acetate buffer of pH 5 in presence of fixed concentration of sulfite ion (10^−3^ M). Equation (1) was used for calculation,

log *K^pot^*_ij_ = log *a_i_* − log *a_j_^Zi/Zj^* (1)

where *a_i_* and *a_j_* are the activities of the primary and interfering ions, respectively, and *z_i_* and *z_j_* are their respective charges.

The obtained results revealed that the CoPC/TDMA^+^/*o*-NPOE-based sulfite sensor exhibited reasonable selectivity towards sulfite ion over most common ions, which was in a good agreement with data obtained by the spectrophotometric measurements. The selectivity coefficients for nitrate, nitrite, chloride, fluoride, azide, sulfate, phosphate, and acetate ranged from 10^−2^ to 10^−3^. Thiocyanate ion, however, significantly interferes but it is fortunate that thiocyanate ion rarely coexists with sulfite in real beverages.

### 2.4. Potentiometric Batch and Flow Injection Analysis (FIA) of Sulfite

Replicate measurements of 1.0 and 10.0 µg/mL internal quality control sulfite sample (IQS) under batch mode of operation and calculation of the student’s (*t* )value at 95% confidence level were made. No statistical difference was detected between the calculated and the theoretical values.

**Figure 5:**
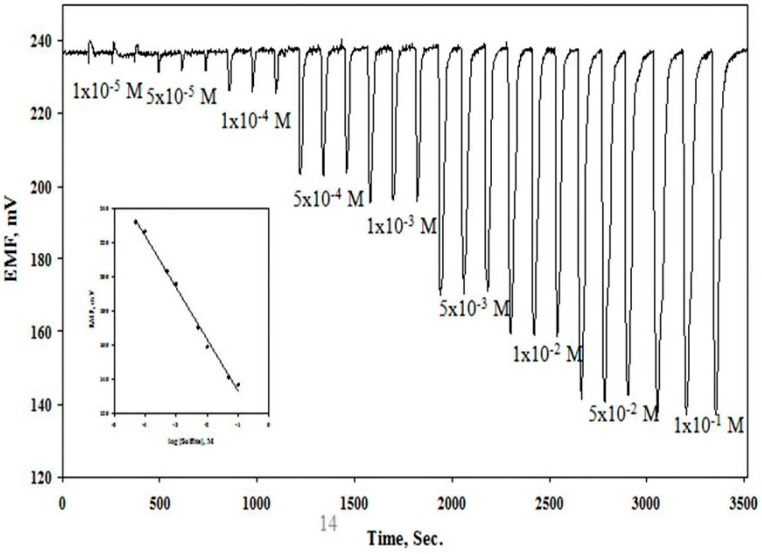
Potentiometric FIA chart for sulfite calibrants using [CoPC/TDMA^+^/*o*-NPOE] PVC membrane.

A flow injection analysis (FIA) of different sulfite calibration solutions using a CoPC/TDMA^+^ based tubular detector was made. The calibration data exhibited good linearity over the concentration range 5.0 × 10^−5^ to 1.0 × 10^−1^ M. The detected low sulfite concentration and wide linear range of measurements permitted accurate monitoring of sulfite in a variety of real beverage samples in the drink industries. Typical FIA signals for different sulfite concentrations are shown in Figure 5. The rate of analysis is 50–60 runs /hour. The technique offers a simple and rapid viable tool for beverage assessment in view of the absence of any commercial sulfite sensor.

### 2.5. Method Validation

Validation of the proposed potentiometric method was performed, using the quality control/quality assurance standards and guidelines [44,45], to verify that the performance characteristics of the method are suitable for sulfite measurement in beverage industry and to provide documented evidence that thedeveloped proceduredoes what it is intended to do. Six batches (six determinations each) covering the concentration range of 1.0 to 300.0 µg/mL sulfite were used for evaluation of the assay range, accuracy, trueness, bias, precision, lower limit of detection, standard deviation, within-day repeatability (*CV_w_*), between-days-variability (*CV_b_*), ruggedness (robustness), and uncertainty.

#### 2.5.1. Data Precision and Accuracy

Repeated measurements of an internal quality control sulfite samples containing 1.0, 10.0, 50.0, 100.0, 200.0, and 300.0 µg/mL sulfite (*n* = 6, each) showed relative standard deviations (*RSD*) in the range ± 0.9 ± 0.1–1.2 ± 0.2%. Theaverage of sulfite results (*X*) and the standard deviation (*S*) were calculated to evaluate the method precision using Equation (2),

Precision, % = (*S/X*) × 100 (2)

where *X* is the mean of test results obtained for the sulfite reference standard sample and *S* is the standard deviation of *X*. Absolute uncertainty is expressed as: *X* sulfite value ± precision.

The degree of agreement or the closeness of test results obtained by the proposed sensor with the true (labeled) sulfite value was measured by spiking (adding) of a reference sulfite samples. The accuracy of the assay method across the dynamic range of the analytical procedure was calculated using Equation (3) and found to be 98.1 ± 0.7–97.3 ± 1.1%,

Accuracy, % = [ (*X_s_* − *X*)/*X_add_*] × 100 (3)

where *X_s_* is the mean result of the spiked sulfite solution, *X* is the mean result of un-spiked sulfite samples, and *X_add_* is the amount of added sulfite standard.

#### 2.5.2. Data Trueness and Bias

Replicate analyses performed on reference sulfite samples (1.0, 10.0, 50.0, 100.0, 200.0, and 300.0 µg/mL sulfite) were also used to determine method trueness and bias according to Equations (4) and (5), respectively.

Trueness, % = (*X/µ*) × 100 (4)

Bias, % = [(*X* − *µ*)/*µ*] × 100 (5)

where *X* is the mean of test results obtained for the reference sample and *µ* is thetrue value of the reference sample. The mean trueness and bias percentages obtained, ranged between 98.0 ± 0.8–97.1 ± 1.2% and 2.0 ± 0.2–3.2 ± 0.8 %, respectively.

#### 2.5.3. Data Repeatability and Reproducibility

The spread of results when a sulfite sample (1.0–300.0 µg/mL) was measured in the same day and on different days to conform the agreement between results obtained with the sulfite reference sample under different conditions with different sensor assembly and pH meters at different times. The reproducibility (*R*) is calculated from the standard deviation data of the results (*s_R_*) (Equation (6)).

*R* = 2.8 × *S_R_* (6)

where 2.8 *=* 2√2 and is derived from the normal or Gaussian distribution; ISO 5725). The data reproducibility within-day and between-days were found to be 0.7 ± 0.2–1.1 ± 0.4% and 0.9 ± 0.2–1.3 ± 0.5%, respectively.

#### 2.5.4. Dynamic Measurement Range and Limit of Detection

The lower limit of sulfite detection (LOD) was calculated according to the IUPAC guidelines [46]. It is equal to the sulfite concentration corresponding to the intersection of the extrapolated linear segment of the calibration graph. The LOD of the method was found to be 1.0 × 10^−6^ M (0.08 µg/mL) and 1.0 × 10^−5^ M (0.8 µg/mL) under batch and flow injection analysis, respectively. The sensor displayed linear dynamic range between 1.0 × 10^−2^–1.0 × 10^−6^ M (800–0.08 µg/mL) and between 5.0 × 10^−5^–1.0 × 10^−1^ M (4–8000 µg/mL) with near-Nernstian slopes of −27.4 ± 0.3 and −23 ± 0.6 mV/decade with batch and flow injection analysis, respectively. All the results of the validation protocol which support the applicability and suitability of the proposed sensor for accurate routine analysis of sulfite ion in various beverages are shown in Table 1.

#### 2.5.5. X-bar and R-Charts

Based on running duplicate measurements for 10 days, control charts (*X* and *R*) were prepared for monitoring 0.5, 5.0, 100.0, 200.0, and 300.0 µg/mL sulfite standard solutions. The results were used to construct both X- and R-control chart. The distribution of measurements and range of the data under investigation indicated that they were in statistical control [44,45].

### 2.6. Potentiometric Determination of Sulfite in Beverages

The levels of sulfite ion in four real beverage samples were potentiometrically measured using CoPC/TDMA^+^/*o*-NPOE-based PVC membrane sensor. The results were compared with data obtained by the standard iodometric method (Table 2). Using direct potentiometry, 1.18–300.0 µg/mL sulfite calibration solutions were measured, each in triplicate, using the sulfite PVC membrane sensor and used for comparison, followed by sample measurements without pretreatment. Potentiometric flow injection analysis, FIA, technique was also used for the analysis. The results obtained under both batch and FIA mode of operation display 98–102% of the results obtained by the standard iodometric method [47].

The color developed by reaction of sulfite with cobalt phthalocyanine reagent in dimethyl sulfoxide solution was also assessed for sulfite quantization. To 0.5 mL of a 0.1 mg/mL solution of CoPC in DMSO 1.0 mL aliquots of sulfite calibration solutions were added in a quartz cuvette. Sulfite calibration solutions were freshly prepared by diluting 30.0, 60.0, 90.0, 120.0, and 150.0 µl of 10^−2^ M stock sulfite solution with de-ionized water to 1 mL. The reagent and sulfite solutions were mixed and the absorbance readings were recorded at 670 nm as a function of sulfite concentration. Application of the method to real beverage samples containing > 80 µg/mL sulfite, showed results within 98–102% of that obtained by the standard iodometric method [47]. These data revealed that the spectrophotometric method is less sensitive compared with the potentiometric sensors and Beer’s law was obeyed over a narrow and relatively higher sulfite concentration range.

## 3. Materials and Methods

### 3.1. Equipment

An Orion double-channel pH/mV meter (Model SA 720, Waltham, MA, USA) equipped with an Orion combination Ross glass pH electrode (Model 81-02) was used for pH adjustments. A cobalt phthalocyanine-PVC-based membrane sensor in conjunction with an Orion Ag/AgCl double junction reference electrode (Model 90–02) was used for potentiometric measurements of sulfite. Potentiometric signals were recorded using a home-made high-impedance data acquisition system connected to a PC through the interface ADC 16 (Pico tech., London, UK) and a PicoLog for windows (version 5.07) software (London, UK) Potentiometric measurements were performed at 25 ± 1 °C.

**Figure 6:**
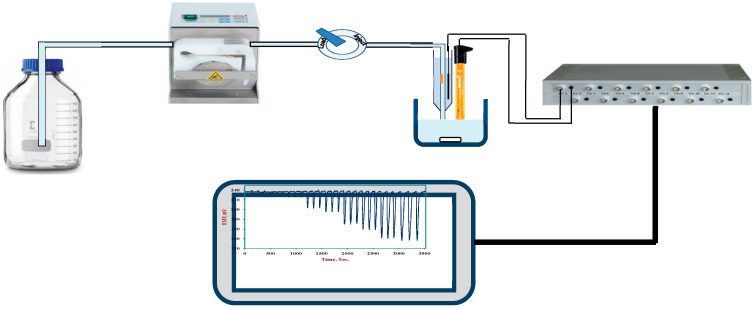
Flow-injection setup: (1) NaCl-acetate buffer reservoir, (2) peristaltic pump, (3) injection valve, (4) cell assembly, (5) double junction reference electrode, (6) high (Z) box, (7) typical flow-transient plot.

A laboratory-made potentiometric flow-through tubular cell was equipped with cobalt phthalocyanine-PVC membrane as previously described [48] and incorporated in a single FIA system, with an Omnifit injection valve and a peristaltic pump (Ismatic, MA, USA) cassette junior model. The potentiometric output was measured with the data acquisition setup. A laboratory-made flow-through tubular potentiometric cell, equipped with CoPC-PVC membrane, was fabricated as previously described [48] and used in a single-channel flow injection system. The cell was assembled and incorporated in a flow injection analysis system. The system consisted of an Ismatic cassette pump (Ismatic, MA, USA) an Omnifit injection valve (Omnifit Cambridge, UK) with sample loop of 20 µL volume. The flow cell and an Orion 90–02 Ag/AgCl double junction reference electrode were placed in a beaker filled with the electrolyte carrier solution. The carrier stream was propelled by peristaltic pump, through Tygon tubing (0.8 mm i.d.) and a mixing coil (15 cm). The potentiometric signals were monitored with the computerized data acquisition system described above. The FIA system setup is shown in Figure 6. Spectrophotometric measurements were carried out using a computer-controlled UV/Vis double-beam spectrophotometer (Shimadzu, model 1601).

### 3.2. Materials and Chemicals

All reagents were prepared from analytical reagent grade chemicals unless otherwise specified and doubly distilled deionized water was used throughout. Cobalt phthalocyanine (CoPC) was obtained from Midcentury (Posen, IL, USA). Tri-dodecylmethyl- ammonium chloride (TDMAC), high molecular weight poly (vinyl chloride) (PVC), M.W. of 100,000, sodium sulfite (Na_2_SO_3_), potassium iodate, potassium iodide, sulfamic acid, sulfuric acid, acetic acid, sodium hydroxide, sodium bicarbonate, sodium chloride, dimethyl sulfoxide (DMSO), dioxane, and sodium chloride (NaCl) were obtained from Aldrich Chemical Co. (Milwaukee, WI, USA). Tetrahydrofuran (THF), freshly distilled prior to use, *o*-nitrophenyloctyl ether (*o*-NPOE), and dioctylphthalate (DOP) were purchased from FlukaChemika-Biochemika (Ronkonkoma, NY, USA).

### 3.3. Preparation of Sulfite PVC Membrane Sensor

The polymeric sensing membrane of the sensor was prepared by dissolving 132 mg PVC, 66 mg of plasticizer, 2 mg of CoPC, and 0.4 mg TDMAC in 2 mL of THF in a glass vial. The membrane cocktail was poured into a glass cup (22 mm i.d.) and covered with a piece of filter paper until complete evaporation of THF. A disk of about 6 mm in diameter was then cut, by the aid of a cork borer, and glued to a piece of Tygon tube (5 mm in inner diameter, 9 mm in outer diameter, and 2 cm length) using THF. The Tygon tube was attached to the electrode glass body, and a 1:1 mixture of 10^−2^ M NaCl solution and 10^−2^ M sodium sulfite solution was used as internal filling solution. An Ag/AgCl electrode was used as internal reference electrode. The potential response of the sensor was measured against an external Ag/AgCl double junction reference electrode.

### 3.4. Sensor Calibration

The sulfite sensor in conjunction with Ag/AgCl double-junction reference electrode was immersed into a 25 mL beaker containing 10 mL of a 1:1 mix of 1.0 × 10^−3^ M NaCl and 1.0 × 10^−3^ M acetate buffer of pH 5. Aliquots (1.0 mL of standard sulfite solutions (1.0 × 10^−6^ to 1.0 × 10^−2^ M) were sequentially added and the potential change after each addition was recorded. A calibration curve was constructed by plotting the potential change against the logarithm of sulfite concentration. The calibration graph was used for subsequent measurement of sulfite levels in real test samples under the same experimental conditions. The sensor was stored in the same solution between measurements.

### 3.5. Potentiometric Batch Determination of Sulfite in Beverages

Sulfite levels in some beverage samples were potentiometrically measured. The standard iodometric method for sulfide assessment was carried in parallel for comparison. The test samples were used without any further treatment. To a 50 mL beaker containing 10 mL of a 1:1 mix of 1.0 × 10^−3^ M NaCl and 1.0 × 10^−3^ M acetate buffer of pH 5, add a 10.0 mL aliquot of the beverage sample and stir. The working and reference electrodes were immersed in the solution. The potential readings were recorded after stabilization and the concentrations of sulfite in the test samples were calculated using the calibration graph. A blank experiment was carried out under the same conditions without the beverage sample.

### 3.6. Potentiometric Flow Injection Analysis (FIA) of Sulfite in Beverages

A flow stream of the carrier solution (1:1 mix of 1.0 × 10^−3^ M NaCl and 1.0 × 10^−3^ M acetate buffer of pH 5) was propelled with a flow rate of 10 mL/min. The carrier solution was allowed to passes through the flow through-cell by means of a peristaltic pump and Tygon tubing. Successive injections of 20 µL aliquots of each sulfite standards (1.0 × 10^−1^–1.0 × 10^−5^ M) were made into the flowing stream. The corresponding signal heights and potential change was measured using a data acquisition system. The mV vs. logarithm sulfite concentration readings of the sulfite standards were recorded and used for construction of a calibration plot. Beverage solutions were similarly injected (in triplicate) in the flowing stream and the average potential readings of three runs was compared with those of the standard solutions. A blank experiment under the same conditions was carried out.

### 3.7. Iodometric Determination of Sulfite in Beverages

A 100.0 mL aliquot of the beverage test solution was transferred to a 250 mL Erlenmeyer flask; add 1 mL of 1:1 sulfuric acid and 0.1 g sulfamic acid crystals. A 1 mL portion of a starch indicator solution was added and titration was conducted with a standard 0.002 M KI/KIO_3_ solution (0.4458 g KIO_3_ + 4.35 g KI + 310 mg NaHCO_3_ in 1 L bi-distilled water). The end point was signaled by the dark purple color due to interaction of the first excess of iodine with starch; 1.00 mL of the titrant ≡ 500 µg sulfite.

## 4. Conclusions

Sulfite additives in real beverage samples are determined using a simple novel potentiometric sensor based on the use of cobalt phthalocyanine (CoPC) ionophore. The sensor consists of poly (vinyl chloride) membrane containing CoPC/TDMAC^+^/*o*-NPOE as an ion recognition sensing material. The method covers the range 1.0 × 10^−6^ to 2.2 × 10^−3^ M (0.08–176 µg/mL) with a low detection limit of 1.0 × 10^−6^ M (0.08 µg/mL), and good selectivity over many other common anions. The performance characteristics of the sensor are validated and proved to be suitable for spectrophotometric covers the linear range 16 to 80 µg/mL. Both methods are used for determination of sulfite in real beverages. The results show good correlation with the standard iodometric method. The proposed method offers many advantages over many of those previously described it needs no prior treatment of the sulfite sample, it has a fast response and is cost-effective, and uses simple equipment.

## Figures and Tables

**Table 1:** Performance characteristics of the [CoPC/TDMA^+^/o-NPOE] sulfite PVC membrane sensor in 10^−2^ M acetate buffer and 10^−3^ M NaCl (pH 5) under batch and FIA modes of operation.

Parameter	Value	
	Batch	FIA
**Calibration range, *M***	1.1 ± 0.1 × 10^−6^–2.2 ± 0.1 × 10^−3^	5.0 ± 0.5 × 10^−5^–1.0 ± 2 × 10^−1^
**Slope, *mV/log [SO_3_]***	−27.4 ± 0.3	−23.7 ± 0.6
**Low detection limit, *M***	1.1 ± 0.1 × 10^−6^	1.1 ± 0.3 × 10^−5^
**Correlation coefficient, *r^2^***	0.9994	0.9965
**Response time for 10^−5^ M, *s***	5 ± 2	50 ± 5
**Recovery time for 10^−5^ M, *s***	10 ± 3	55 ± 5
**Working acidity range, *pH***	5–7	5–7
**Accuracy, *%***	98.1 ± 0.7	97.3 ± 1.1
**Trueness, *%***	98.0 ± 0.8	97.1 ± 1.2
**Bias, *%***	2.0 ± 0.2	3.2 ± 0.8
**Within-day Repeatability, *CV_w_*%**	0.7 ± 0.2	1.1 ± 0.4
**Between days- variation, *CV_b_*%**	0.9 ± 0.1	1.3 ± 0.5
**Relative Standard deviation, *%***	0.9 ± 0.1	1.2 ± 0.2
**Precision, *%***	0.7 ± 0.2	0.8 ± 0.4

**Table 2:** Determination of sulfite in some beverage samples using potentiometry with the sulfite sensor under batch and FIA modes of operation, spectrophotometry with cobalt phthalocyanine and the standard iodometric method.

Sample	Sulfite, µg/ mL			
	Potentiometry, (Sulfite Sensor)		Spectro- Photometry	Iodometry, [47] (Standard Methods)
	Batch	FIA		
**Beer,(Barq’s), USA**	10.9 ± 0.6	10.4 ± 0.8	-	11.1 ± 0.7
**Non-alcoholic Malt Beverage, (Birell), Egypt**	8.1 ± 0.7	7.9 ± 0.8	-	8.2 ± 0.8
**Non-alcoholic Malt Beverage, (Barbican), USA**	9.1 ± 0.6	9.6 ± 0.8	-	9.1 ± 0.8
**White Sparkling Grape Drink, (Carl Yong), Germany**	277.2 ± 0.6	281.1 ± 0.9	275.3 ± 0.7	278.4 ± 2.3
**White Vinegar, (Heinz), Egypt**	87.0 ± 0.6	89.2 ± 1.7	86.9 ± 1.9	88.3 ± 2.3
**Sugar Lump, (National Sugar Co.), Egypt**	244.2 ± 0.6	250.3 ± 1.3	252.2 ± 0.8	247.2 ± 2.6
